# Blood neutrophil activation markers in severe asthma: lack of inhibition by prednisolone therapy

**DOI:** 10.1186/1465-9921-7-59

**Published:** 2006-04-06

**Authors:** Bhupinder S Mann, Kian Fan Chung

**Affiliations:** 1 National Heart & Lung Institute, Imperial College & Royal Brompton Hospital London, UK

## Abstract

**Background:**

Neutrophils are increased in the airways and in induced sputum of severe asthma patients. We determined the expression of activation markers from circulating neutrophils in severe asthma, and their supressibility by corticosteroids.

**Methods:**

We compared blood neutrophils from mild, moderate-to-severe and severe steroid-dependent asthma, and non-asthmatics (n = 10 each). We examined the effect of adding or increasing oral prednisolone (30 mg/day;1 week).

**Results:**

Flow cytometric expression of CD35 and CD11b, but not of CD62L or CD18, was increased in severe asthma. F-met-leu-phe increased CD11b, CD35 and CD18 and decreased CD62L expression in all groups, with a greater CD35 increase in severe asthma. In severe steroid-dependent asthma, an increase in prednisolone dose had no effect on neutrophil markers particularly CD62L, but reduced CD11b and CD62L on eosinophils. Phorbol myristate acetate-stimulated oxidative burst and IL-8 release by IL-1β, lipopolysaccharide and GM-CSF in whole blood from mild but not severe asthmatics were inhibited after prednisolone. There were no differences in myeloperoxidase or neutrophil elastase release from purified neutrophils.

**Conclusion:**

Because blood neutrophils in severe asthma are activated and are not inhibited by oral corticosteroids, they may be important in the pathogenesis of severe asthma.

## Background

Asthma is characterised by reversible airways obstruction, bronchial hyperresponsiveness, and chronic inflammation of the bronchial mucosal lining. Inhaled corticosteroids, particularly in combination with long-acting β-agonists, are efficient in reducing symptoms and exacerbations. However, a proportion of patients with asthma are not controlled and have persistent symptoms, recurrent exacerbations and/or persistent airflow obstruction despite using high doses of inhaled corticosteroids, and often oral corticosteroids, and long-acting β_2_-agonist bronchodilators [1]. This group of patients often termed severe asthma or therapy-resistant asthma forms a distinct category of asthma [2,3]. There may be pathophysiological mechanisms that are unique for severe asthma compared to mild/moderate asthma [4]. The neutrophil may be an important inflammatory cell that contributes to the pathophysiology of severe asthma since increased neutrophilic inflammation measured in induced sputum and in the bronchial submucosa has been reported in such patients [5-7] Increased neutrophilic inflammation has also been observed under other asthmatic circumstances such as in patients who have died during a sudden-onset attack and in patients ventilated following an acute severe exacerbation [8,9]. In addition, increased neutrophils have been observed in airway submucosal glands in patients who have died of asthma [10].

Neutrophils may therefore be involved in the pathogenesis of asthma through their activation [11-13]. Neutrophil migration from the circulation into an area of inflammation involves regulated expression of leucocyte surface adhesion molecules. L-selectin (CD62L) is important in the initial attachment of leucocytes to endothelium, and is rapidly shed after neutrophil activation by proteolytic cleavage [14]. This is followed by tight adhesion and transendothelial migration of granulocytes mediated by β2 integrins, such as CD11b (CR3, or Mac1), one of 3 α-chain molecules, and CD18, a common β-chain. Neutrophil activation is associated with upregulation of CD11b and CD18 from intracellular storage pools, and shedding of CD62L. Neutrophils are also a potential source of inflammatory mediators such as leukotriene B4 (LTB4), and platelet-activating factor (PAF), of enzymes such as neutrophil elastase, myeloperoxidase and matrix metalloproteinases (MMP-9), of reactive oxygen species, and of cytokines such as TNFα and IL-8 [15]. Peripheral blood neutrophils and eosinophils may be activated or primed in the circulation of mild asthmatics with regards to superoxide production, chemotaxis and increased expression of surface molecules on neutrophils such as CD35, which mediates the binding and phagocytosis of C3b-coated particles and immune complexes [16]. Neutrophils have been implicated in the development of bronchial hyperresponsiveness [17]. Neutrophil elastase has the capacity to induce mucus gland hyperplasia and mucus secretion) [18] and proliferation or apoptosis of airway smooth muscle cells [19,20]. Little is known about the activation status and the role of the circulating neutrophil in severe asthma.

Glucocorticoids, an important treatment for asthma, exert inhibitory effects on neutrophil activation and functions such as chemotaxis, free radical generation, and adhesion [21-24]. They also inhibit neutrophil apoptosis, while decreasing eosinophil survival by increasing eosinophil apoptosis [25]. Treatment of mild asthmatics with prednisolone induces an increase in neutrophils in the airways mucosa [26], but in patients with chronic obstructive airways disease, corticosteroids have very little effect on airway inflammation [27,28]. In the present study, we have investigated the expression of the cell-surface molecules CD11b, CD62L, CD18 and CD35 on resting and stimulated neutrophils, together with respiratory burst activity, myeloperoxidase and neutrophil elastase release, and IL-8 cytokine levels from blood cells in different categories of patients with asthma. We have categorised our asthmatics into a group of severe corticosteroid-dependent asthmatics on both inhaled and oral corticosteroid therapies, compared to a group of moderate-to-severe asthmatics only on inhaled corticosteroid therapy and a group of mild asthmatics. We also studied the effect of oral prednisolone or of an increase in prednisolone dose on these neutrophil parameters in the severe and in the mild asthma patients.

## Materials and methods

### Subjects

We studied 10 patients with mild asthma, 10 with moderate-to-severe asthma, 10 severe steroid-dependent asthmatics, and 10 healthy non-asthmatic volunteers (Table 1). All asthmatics had a history of episodic dyspnoea, wheezing and reversible airway obstruction. Asthma was diagnosed either on the basis of bronchial hyperresponsiveness to methacholine with a PC_20 _< 4 mg/ml when baseline FEV_1 _was > 70%, or by demonstrating > 15% improvement in baseline FEV_1 _following inhalation of salbutamol (400 μg) when the FEV_1 _was < 70%. Normal subjects had no chest symptoms, with FEV_1 _> 90% predicted. All subjects were non-smokers and have never smoked previously, and asthmatics were stable with no changes in asthma symptoms and medication for at least one month, except for the use of short-acting inhaled β_2 _agonists. No subject had a history of an upper respiratory tract infection within the previous 6 weeks.

**Table 1:** Clinical characteristics of controls and asthmatic patients

	**Mild asthma**	**Moderate-to-severe asthma**	**Severe steroid-dependent asthma**	**Non-asthma controls**
n	10	10	10	10

Gender M:F	2:8	4:6	4:6	7:3

Mean age (years)	35.8	47.7	50.9	36
[range]	22–49	31–73	45–66	32–45

FEV1 (% predicted)^1^	95.8 ± 3.3	66.7 ± 5.0	51.4 ± 3.7	95
[range]	75–112	38.5–85	30–70.7	92–98

Inhaled corticosteroid dose (BDP equivalent μg/day)^1^	0	1,900 ± 100	2,000 ± 0	0

Atopy*	7	10	5	0

Exhaled NO (ppb)	27.1	46.9	30.8	ND
[range]	6.2–94	9.2–102	10.4–65.2	

M: male; F: female; NO: nitric oxide; ND: not determined.

*: Atopy defined as positive skin prick test to 2 common aeroallergens

^1^: Data shown as mean ± SEM

Mild asthmatics had symptoms less than once a week, requiring only a short-acting inhaled β_2_-agonist as needed. Moderate-to-severe asthmatics had asthma symptoms requiring daily use of inhaled short-acting β_2_-agonists and inhaled corticosteroids (beclomethasone dipropionate 1,000 to 2,000 μg daily or equivalent) via a metered-dose inhaler, but not requiring chronic oral prednisolone therapy. Severe steroid-dependent asthmatics were defined as having persistent symptoms with frequent exacerbations, limited physical activity and frequent night-time symptoms requiring high dose inhaled corticosteroids (beclomethasone dipropionate, 2000 μg/day or equivalent), and with maintenance oral prednisolone (> 5 mg per day for at least one year). The mean maintenance dose of oral prednisolone was 14 mg/day (range 10–20 mg/day).

Atopic status was evaluated by skin prick tests to *Dermatophagoides pteronyssinus*, mixed grass pollen, and cat hair. FEV_1 _was measured with a calibrated, dry-wedge spirometer (Vitalograph, Buckingham, UK.). All patients gave informed consent, and the study was approved by the Royal Brompton Hospital Ethics Committee.

### Effect of prednisolone

The effect of prednisolone on neutrophil function in peripheral blood was determined by treating mild asthma patients with prednisolone 30 mg/day for 1 week, and steroid-dependent patients with a dose of 30 mg/day added to their usual maintenance dose for one week. Blood samples were obtained before and on the day after the seventh dose of prednisolone had been taken. The study was not blinded or placebo-controlled, but the person analysing the cells ex-vivo was blinded to the treatment received by the asthmatics.

### Surface molecule expression

Fluorescein isothiocyanate (FITC)-conjugated monoclonal antibodies directed against CD11b, CD62L (L-selectin), CD35 and CD18, along with the appropriate isotype controls (Serotec Ltd, Oxford, UK) and phycoerythrin (PE)-conjugated CD16 monoclonal antibody (Becton Dickinson, Oxford, UK) were added to heparinised whole blood (100 μl) at 4°C for 30 min in the dark. Cells were washed with phosphate-buffered saline (PBS, Gibco, Invitrogen, UK) containing 0.5% bovine serum albumin (BA, Sigma Aldridge, Poole, UK) and 0.5% sodium azide (Sigma Aldridge, Poole, UK). After centrifugation (5 minutes; 1800 rpm) the supernatant was discarded and 2 ml of freshly-prepared Erythrolyse (Serotec Ltd, Oxford, UK) were added (10 min; room temperature). Cells were then washed with 2 ml PBS-BA and centrifuged as above and resuspended in 0.5 ml of 0.5% paraformaldehyde (Sigma Chemical Co, Poole, UK).

### Stimulation of whole blood

Blood was incubated with N-formyl-methionyl-leucine-phenylalanine (FMLP 10^-7^M, Sigma Aldridge, Poole, UK) for 10 min at 37°C. The reaction was stopped by adding cold PBS solution. The tubes were then centrifuged for 5 min at 1800 rpm and the supernatant discarded. Blood was then incubated with conjugated monoclonal antibodies.

### Flow cytometry

Flow cytometry was performed using a FACScan analyser (Becton Dickinson, Oxford, UK) with Argon-ion laser. Fluorescence gain was adjusted using a gate drawn around the granulocyte population on an unstained lysed whole blood sample, and then placing this population in the first log decade. Compensation was set by using blood incubated with either FITC- or PE-labelled monoclonal antibodies in order to correct for spectral overlap. As an instrumental control measure, CaliBRITE beads (Becton Dickinson) were used to ensure the correct alignment and calibration of the flow cytometer. Ten thousand events were collected from each sample. We used the method described by Gopinath et al for identification of neutrophils and eosinophils in lysed whole blood using side scatter and CD16 negativity [29]. Granulocytes were discriminated from other cell populations by their characteristic high side and forward light scatter. A gate was drawn around the granulocytes which was reflected into a dot-plot of side-scatter and CD16, showing neutrophils as CD16-positive cells with eosinophils as CD16-negative cells with high side scatter. Purity of the neutrophil and eosinophil populations determined was close to 99% on cytospin preparations, as previously described. A further gate was then drawn around the neutrophil (CD16^+^) or eosinophil (CD16^-^) populations and reflected into a histogram so that cells can be labelled with a further fluorochrome (eg. CD11b or CD18). Results were recorded as mean fluorescence intensity (MFI) which represents the cell surface receptor density.

### Oxidative burst and elastase measurements

Respiratory burst was measured according to the method of Rothe and Valet [30]. Briefly, 3 ml of heparinised blood was layered onto 3 ml of Ficoll Hypaque medium (Amersham Biosciences, UK) and left at room temperature for 45 min. The supernatant (800 μl) was then removed and transferred to an Eppendorf tube and stored on ice. Hank's balanced salt solution (HBSS, 1 ml; Sigma Aldridge, Irvine, UK) was added to 20 μl of cell suspension and 10 μl of dihydrorhodamine 123 (DHR123, 1 × 10^-3^M, Molecular Probes, Upssala, Sweden), and the cells were incubated for 5 min at 37°C. Phorbol myristate acetate (PMA, Sigma Aldridge, Poole, UK; final concentration 1 μM) was added for 20 min at 37°C. Reagent blank tubes were prepared by adding only PBS to the cell suspension, while resting cells were initially incubated with PBS, followed by the addition of dihydrorhodamine.

After the final incubation, samples (250 μl) were analysed immediately on the FACS scanner. The FACS machine was set up so that unstimulated cells were placed in the first log decade. DHR 123 reacts with hydrogen peroxide as a result of the respiratory burst to give rhodamine which emits a green fluorescence which was measured on the FL1 channel at a wavelength of 530 nm.

Elastase was also measured according to the method described by Rothe and Valet [31]. HBSS supplemented with 10 mM HEPES (Sigma Aldridge, Poole, UK; 1 ml) was added to 20 μl cell suspension, and 1 μl (N-benzyloxycarbonyl-Ala-Ala)_2_-rhodamine 110 (Molecular probes, Upssala, Sweden; final concentration 4 μM). The samples were analysed on the flow cytometer after 20 min.

### Whole blood culture

Venous blood was collected in heparinized tubes and diluted 1:10 with RPMI 1640 (Gibco, Invitrogen, UK) containing 10% fetal calf serum (FCS), 100 U/ml penicillin, 100 μg/ml streptomycin and 2 mM L-glutamine. The blood was distributed in 1 ml wells and the plates were incubated at 37°C with 5% CO2 for 24 hours. In addition, in duplicate wells, diluted blood was stimulated with IL-1β (10 ng/ml final concentration), lipopolysaccharide (LPS 10 ng/ml), and GM-CSF (1 ng/ml) prior to incubation. The plates were then centrifuged at 1800 rpm for 5 minutes, following which the supernatants were removed and frozen at -70°C until analysed.

### Myeloperoxidase release from purified neutrophils

Granulocytes were isolated by Ficoll-Hypaque gradient centrifugation of heparinised whole blood (20 ml). After centrifugation (2000 rpm; 20°C; 20 min), all the layers above the red cells were removed and 10 ml PBS and 3 ml 3% Dextran T-500 (Pharmacia Biotech, Uppsala, Sweden) were added. The supernatant was centrifuged and red cells were lysed by adding 2 ml 10% HBSS in distilled water for 20 seconds, following which excess HBSS (minimum 12 ml) was added and the contents of the tube mixed and centrifuged at 1800 rpm for 5 minutes. The cells were washed twice in HBSS (Ca^++^- and Mg^++^-free). Granulocytes (1 × 10^6^/ml) were then added to 1 ml wells of a 48-well plate and incubated with IL-β (10 ng/ml) or PBS for 30 min, and incubated at 37°C under 5% CO2. After each time-point, the plates were centrifuged (1800 rpm; 5 min) following which the supernatant was withdrawn and stored at -70°C until analysed for MPO levels.

### Enzyme-linked immunosorbent assay (ELISA)

IL-8 (duoset, R&D systems, Oxon, UK) and neutrophil myeloperoxidase (MPO; Calbiochem, San Diego, USA) concentrations were measured in the culture supernatants with specific immunoassay kits according to the manufacturer's instructions. The detection limit was 31.2 pg/ml for IL-8 and 1.6 ng/ml for MPO.

### Statistical analysis

Unless otherwise stated, data are expressed as mean ± standard error of the mean (SEM). Differences in surface marker expression between groups were evaluated using Kruskall Wallis test. Differences between each of the groups and controls were evaluated using the Mann-Whitney U-test, with application of Bonferroni correction. The effects of prednisolone therapy or of the stimulation with various stimulants *in vitro *were analysed by the Wilcoxon signed-rank test. A *p *value of 0.05 or less was considered to be statistically significant.

## Results

### Surface molecule expression on lysed whole blood

**Figure 1:**
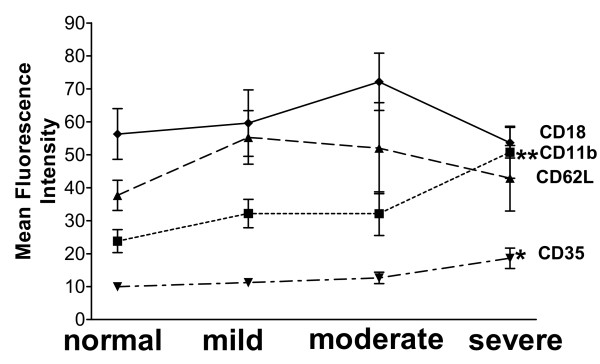
Mean fluorescence intensity (MFI) of CD11b, CD62L, CD35, and CD18 expression on neutrophils from lysed whole blood from 10 normal, 10 mild, 10 moderate-to-severe and 10 severe steroid-dependent asthma patients. There was increased expression of CD11b and CD35 in severe asthmatics. Data shown as mean ± SEM. * p < 0.05; ** p = 0.01.

In unstimulated blood, there was an increased expression of CD35 and CD11b on neutrophils in patients with severe steroid-dependent asthma compared to mild and moderate-to-severe asthmatics, and non-asthmatic volunteers (p < 0.05). There were no significant differences in the expression of CD62L or CD18 between the groups (Fig 1).

**Figure 2:**
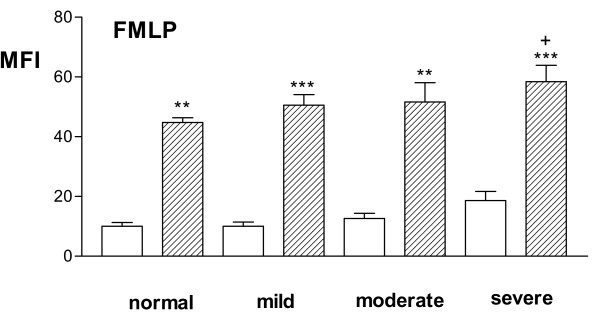
Mean fluorescence intensity of CD35 expression on neutrophils from 10 normal, 10 mild, 10 moderate-to-severe and 10 severe steroid-dependent asthma patients before (open bars) and after (striped bars) stimulation with f-met-leu-phe (FMLP). **p < 0.01, *** p < 0.0001, compared to resting levels; +p = 0.02 compared to level from FMLP-stimulated blood from normal volunteers.

Stimulation of blood with FMLP increased the expression of CD11b, CD35, and CD18 surface molecules on gated neutrophils in all groups of patients; there was concomitantly a significant decrease in CD62L in all groups. The increase in CD35 expression after stimulation with both FMLP was greater in severe asthmatics compared to that seen in non-asthmatic volunteers (p = 0.02; Fig 2), but was not significantly different compared to the other asthmatic groups.

**Figure 3:**
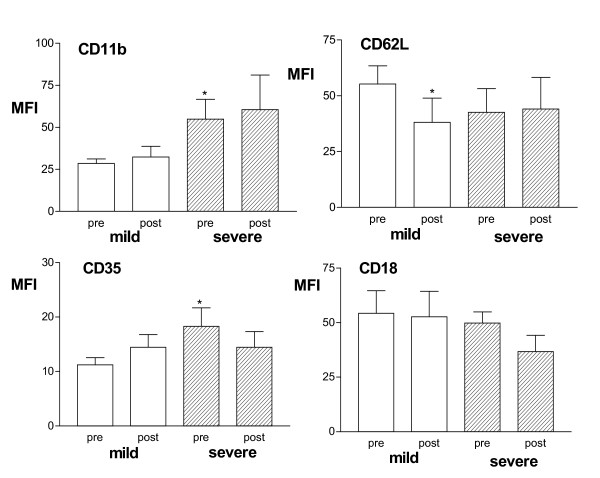
Mean fluorescence intensity (MFI) of CD11b, CD62L, CD35 and CD18 expression on blood neutrophils in 10 mild and 9 severe steroid-dependent asthma before (pre) and after (post) a course of prednisolone or of increased prednisolone dose (30 mg/day for one week). CD62L expression decreased in mild asthmatics following prednisolone, but there was no effect in patients with severe asthma. *p < 0.05 compared to pre-values in mild asthma group.

**Figure 4:**
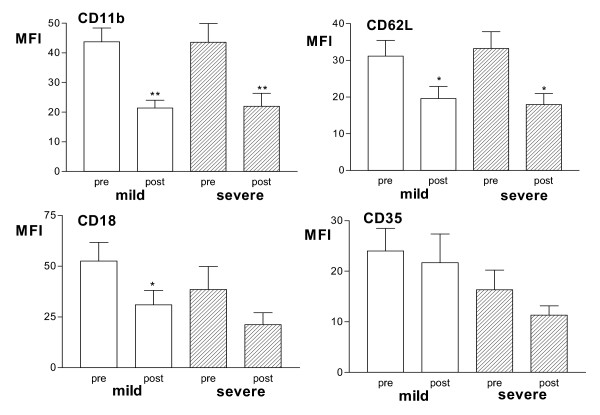
Mean fluorescence intensity (MFI) of CD11b, CD62L, CD35 and CD18 expression on blood eosinophils in 10 mild and 9 severe steroid-dependent asthmatics before (pre) and after (post) a course of prednisolone. *p < 0.05; **p < 0.01 compared to within-group pre-values.

Prednisolone had no effect on the expression of CD11b, CD62L, CD35 or CD18 on neutrophils from the 10 severe corticosteroid-dependent (Fig 3). In the 10 mild asthmatics, there was a significant decrease in the level of CD62L after prednisolone (Fig 3); this decrease was from a higher baseline expression of CD62L, though not statistically different from neutrophils from severe asthma patients. In contrast, no difference was seen in the baseline expression of the above surface molecules on peripheral blood eosinophils between the patient groups (data not shown). Following prednisolone, however, there was a significant reduction in the expression of CD11b and CD62L on eosinophils in both mild and severe patients, and a reduction in CD18 in mild asthmatics (Fig 4).

### Oxidative burst in peripheral blood neutrophils

**Figure 5:**
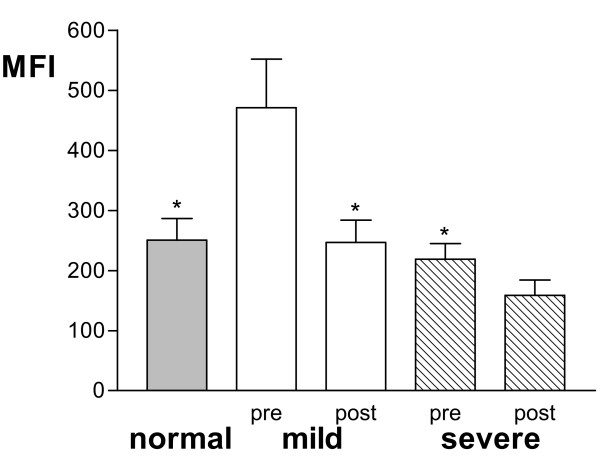
Mean fluorescence intensity (MFI) of oxidative burst from neutrophils stimulated with phorbol myristate acetate (PMA) in 9 normal volunteers (normal) or in 9 mild and 9 severe steroid-dependent asthmatics before (pre) and after (post) prednisolone. Data shown as mean ± SEM. * p < 0.01 compared to pre-values in mild asthma group.

PMA stimulated the oxidative burst in granulocytes in a dose-dependent manner, with maximal stimulation achieved after 20 min (data not shown). The oxidative burst in mild asthmatics was greater than that in normal subjects (p = 0.02), while similar levels of oxidative burst were seen between normal subjects and severe asthma patients (Fig 5). After prednisolone, the oxidative burst was reduced in mild asthma patients but not in severe patients.

### Release of IL-8 from whole blood culture

**Figure 6:**
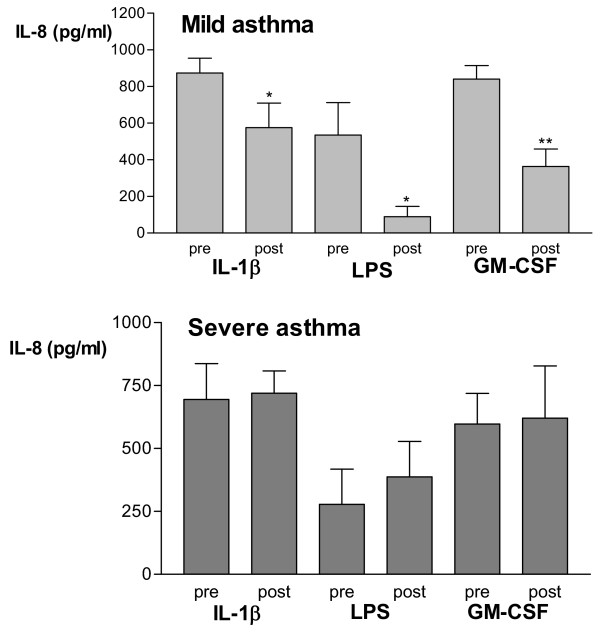
IL-8 release from whole blood after stimulation with IL-β, lipopolysaccharide, and GM-CSF from 7 mild (upper panel) and 7 severe steroid-dependent (lower panel) asthmatics, measured before (pre) and after (post) prednisolone. Data shown as mean ± SEM. *p < 0.05, **p < 0.01 compared to corresponding pre-values for each stimulus.

In unstimulated blood, IL-8 was not detectable in serum in patients with mild or severe asthma but after 24 hours, IL-8 was detectable. IL-8 levels from mild asthmatics were not significantly elevated after stimulation with IL-1β, LPS, and GM-CSF compared to severe asthmatics. In the mild group, however, there was a significant decrease in IL-8 production induced by IL-1β, LPS and GM-CSF following prednisolone therapy; this effect of prednisolone was not seen in the severe group (Fig 6). There were no significant differences in the release of IL-8 in the pre-prednisolone blood between the patients mild and severe asthma.

### Neutrophil elastase and myeloperoperoxide release from neutrophils

Flow cytometric analysis of whole blood showed no difference in the baseline level of neutrophil elastase between mild or severe asthmatics, and there was no significant effect of prednisolone on these levels. No significant difference was seen in the baseline or IL-β-stimulated MPO levels in purified blood neutrophils between the mild and severe asthmatics. There was no significant effect of prednisolone on the MPO levels in either group.

## Discussion

We determined whether circulating neutrophils in patients with severe asthma exhibited evidence of activation in terms of expression of surface markers or mediator release. CD35 and CD11b expression was higher on resting peripheral blood neutrophils in severe steroid-dependent asthmatics compared to normal and mild asthmatics, suggesting that neutrophils are in a more activated state in the circulation of severe asthmatics. In addition, CD35 expression on neutrophils from severe asthmatics was increased further above that seen in normal patients following stimulation of whole blood with fMLP. No difference in the expression of these surface molecules was seen on eosinophils in any of the groups.

In previous studies of neutrophil surface marker expression in asthma, no differences in expression of CD11b and CD62L had been observed compared to non-asthmatic controls [32], although an elevation in CD35 had been reported [16]. These are in contrast to the current results showing no significant differences in expression of CD35, CD11b and CD62L in mild and moderate asthmatics compared to normal volunteers. However, in this first study of blood neutrophils in severe asthma, we found an increase in CD35 and CD11b expression in patients with severe steroid-dependent asthma. Stimulation of neutrophils with fMLP increased expression of CD11b on neutrophils and eosinophils to a larger extent in children with mild asthma than in controls [16]. We found no such differences in our adults with mild asthma, but CD35 was slightly more inducible on neutrophils from severe steroid-dependent asthmatics. The discrepancies between these studies are not clear, but may relate to the age of the patients, to methodological differences in assaying these markers and to the grading of severity of the patients.

The cause of the increase in CD35 and CD11b expression on neutrophils from patients with severe asthma is unclear. One confounding factor is that the patients with severe asthma were on chronic oral prednisolone therapy which can induce blood neutrophilia attributable to bone marrow release of neutrophils, and also to mobilisation of a marginating blood pool [33,34]. Prednisolone treatment of mild asthmatics did not result in an increase in CD35 or CD11b expression, suggesting that the recruitment of new neutrophil pools may not be the cause of the increase seen in severe asthma. However, an inhibition in neutrophil CD62L expression by corticosteroids as observed in mild asthmatics has been attributed to a direct inhibition of CD62L expression in the maturation pool of bone marrow neutrophils [35]. It is likely that the increase in CD35 and CD11b may be a specific abnormality of circulating neutrophils in severe asthma. An intriguing feature is that the enhanced expression of CD11b was not accompanied by a reciprocal increase in the CD18 expression, since CD11b and CD18 constitute the Mac-1 integrin. On the other hand, it is also possible that there is enhanced shedding of CD18 or a redistribution of CD18 complexes with the CD11 family.

One of the problems of doing comparative studies of these groups of asthmatic patients separated according to disease severity is that the severe group is usually on chronic prednisolone therapy and it is difficult to exclude the effects of such therapy on neutrophil function in the severe asthma group as accounting for any observed differences. For this reason, we have studied the effect of a course of high dose prednisolone on blood neutrophil function, albeit for only one week.

We found no effect of prednisolone on the expression of CD11b, CD62L, CD35 or CD18 in severe asthma, while, in mild asthma, prednisolone caused a significant decrease in CD62L expression without affecting the expression of CD35, CD11b and CD18. Thus, while the expression of CD62L by prednisolone was suppressed in mild asthma, this was not the case in severe asthma. Our observation in mild asthma patients is consistent with two reports of reduction in CD62L expression following intravenous dexamethasone in normal individuals [36,37]; similarly, high doses of corticosteroids decreased CD62L and CD11b in patients with multiple sclerosis and in patients undergoing cardio-pulmonary bypass [38,39]. The reduction of surface molecule CD62L expression by glucocorticoids may occur on segmented neutrophils within the maturation pool of the bone marrow but not on neutrophils already in the circulation [35]. This may be one mechanism of the anti-inflammatory action of glucocorticoids in mild asthma, whereby the initial weak binding of leucocytes to the endothelium mediated by L-selectin molecules such as CD62L is reduced. By contrast, the lack of inhibitory effect of corticosteroids on CD62L in severe asthmatics may allow granulocyte recruitment into inflammatory sites to continue. This lack of inhibitory effect appears to be selective for neutrophils since surface molecule expression of CD62L on eosinophils from both mild and severe patients were reduced by prednisolone to a similar extent. Thus, in severe asthma, blood eosinophils were sensitive while neutrophils appear to have lost their sensitivity to the effects of increased oral dose of prednisolone.

In addition to the loss of the effect of prednisolone on neutrophil surface markers in severe asthma, there was also lack of inhibition on the stimulated release of IL-8, and oxidative burst. Levels of IL-8 after whole blood culture were not significantly different between mild and severe steroid-dependent asthmatics, but IL-8 levels from severe patients were less readily suppressed after a course of prednisolone compared to mild asthmatics. It must be noted that although neutrophils stimulated with IL-1β, LPS and GM-CSF may be an important source of IL-8, other cells such as monocytes may also be contributory. We conclude that either of these cells were likely to represent the site of reduced corticosteroid responsiveness seen in the blood samples of patients with severe asthma.

The oxidative burst activity of neutrophils was greater in mild asthmatics compared to normal patients and severe asthma patients. Furthermore, prednisolone decreased oxidative burst activity in neutrophils from mild asthmatics, as reported previously in isolated bovine neutrophils [40], but there was no significant effect in the severe patients. Prolonged corticosteroid therapy may lead to inhibition of neutrophil superoxide production, possibly through inhibition of NADPH oxidation or impairment of other transduction mechanisms in the respiratory burst [23]. It is possible that the loss of suppressibility of the oxidative burst to prednisolone in severe asthma could be due to the reduced basal release possibly induced by chronic prednisolone therapy. Examination of the effect of prednisolone in normal volunteers in whom the baseline respiratory burst is similar to that of severe asthma patients would resolve the issue as to whether suppression is possible.

In comparison to whole blood release, when neutrophils were purified and studied *in vitro*, no difference was seen in the basal or IL-1β stimulated myeloperoxidase release from purified granulocytes between normal, mild, or severe asthmatics. The isolation procedure itself may alter the neutrophil to such an extent as to abolish any differences in activation. Monocytes are a source of myeloperoxidase but we have not studied monocytes in this study. No difference was seen in peripheral blood neutrophil elastase levels between mild and severe asthmatics using a flow cytometric method of analysis. Increased levels of neutrophil elastase have been reported in sputum from asthmatics with an exacerbation due to a respiratory tract infection [41], and in patients in status asthmaticus undergoing mechanical ventilation in the absence of infection [8,9].

What is the relevance of our findings on circulating blood neutrophils and on the pathophysiology of the airways of severe asthma? The lack of inhibition of CD62L expression and of the release of reactive oxygen species and IL-8 by prednisolone may explain why there may be ongoing inflammation and asthma in patients with severe asthma. Heightened expression of CD62L may increase the ability of the neutrophil to transmigrate from the circulation into the airway submucosa. Interestingly, the ability of prednisolone to suppress the eosinophil expression of CD11b and CD62L in severe asthma would indicate that eosinophils may be less likely to contribute to the pathophysiological changes in the airways. The lack of inhibition of IL-8 release would also lead to increased neutrophilic inflammation since IL-8 is considered to be an important molecule for the development of neutrophuilic inflammation in asthma. Concentrations of IL-8 are correlated with neutrophil accumulation in asthmatic airways [7], and IL-8 is increased in bronchoalveolar lavage fluid and serum of asthma patients [42,43]. A recent study suggested that neutrophils that have migrated to IL-8 may subsequently cause eosinophils to accumulate to the asthmatic airways [44].

Why is the blood neutrophil less responsive to corticosteroids? Previous studies on circulating blood cells have focused on the peripheral blood mononuclear cell as being the site of corticosteroid resistance [45,46]. Decreased binding affinity of the glucocorticoid receptor (GR), increased expression of the alternatively-spliced variant GR-β, increased binding of the activated GR receptor to pro-inflammatory transcription factors such as AP-1 and reduced recruitment of histone deacetylase have all been implicated as the cellular basis for this resistance to corticosteroids [45,47-49]. High levels of GR-β have been reported in neutrophils, and these may be upregulated by IL-8 [50].

## Conclusion

The circulating neutrophil in severe asthma shows evidence of activation, as measured by the increased expression of the adhesion molecules, CD11b and CD35, and of resistance to the effects of prednisolone. The neutrophil may be an important contributor to the inflammatory process in severe asthma, and further studies are needed on lung neutrophils to support this notion. The mechanism of loss of corticosteroid inhibition of neutrophils in severe asthma is not known and deserves further study.

## Competing interests

The author(s) declare that they have no competing interests.

## Authors' contributions

KFC designed the project, BSM carried out all the studies and both wrote up the manuscript.
